# Vascular Endothelial Cell Function in Catastrophic Antiphospholipid Syndrome: A Case Report and Review of the Literature

**DOI:** 10.1155/2013/710365

**Published:** 2013-05-15

**Authors:** B. Routy, T. Huynh, R. Fraser, C. Séguin

**Affiliations:** ^1^Division of Haematology, Department of Medicine, McGill University Health Center (MUHC), 1650 Cedar Avenue, Montreal, QC, Canada H3G 1A4; ^2^Division of Cardiology, Department of Medicine, McGill University Health Center (MUHC), 1650 Cedar Avenue, Montreal, QC, Canada H3G 1A4; ^3^Department of Pathology, McGill University Health Center (MUHC), 1650 Cedar Avenue, Montreal, QC, Canada H3G 1A4

## Abstract

Catastrophic antiphospholipid syndrome (CAPS) is a rare autoimmune condition, which has been associated with a high mortality rate. However, with current management that includes a combination of anticoagulation, glucocorticoid administration, and plasma exchange, mortality rate has declined. Despite survival improvement with new generation immunosuppressive agents, their mechanisms of action are poorly defined, and CAPS is still considered a high-risk complication in patients known with antiphospholipid antibody syndrome. Herein, we present a case of a 79-year-old male who presented with a myocardial infarct and renal failure secondary to CAPS following a splenectomy for immune thrombocytopenia. Regardless of rapid combination of first-line treatment and rituximab therapy, the patient developed lethal cardiogenic shock secondary to mitral valve papillary muscle necrosis. Discussion of the pathophysiology and avenues of future therapies in CAPS are reported.

## 1. Introduction

Antiphospholipid syndrome (APS) is characterized by the presence of antiphospholipid antibodies in patients who have a history of thrombosis and/or fetal loss. It is an autoimmune disease with a misleading name because the pathologic auto-antibodies are directed against the plasma protein *β*(2)-glycoprotein I and not against phospholipids [1].

Exceptionally, patients with APS may develop a “catastrophic” variant [2]. Catastrophic Antiphospholipid Syndrome (CAPS) is defined as a life-threatening condition with widespread small vessel thromboses in a patient with laboratory confirmation of antiphospholipid antibodies [3]. First-line therapy includes a combination of anticoagulants, glucocorticoids, immunoglobulins, and plasma exchange [4–6]. Despite recent survival improvement related to the usage of newer immunosuppressive agents such as rituximab, CAPS still has an estimated 33.3% mortality rate [7]. Cardiac complications are the second most common cause of death after cerebral vascular disease [8–10]. The cardiac manifestations include valvular endocarditis and microvascular thromboses [9].

We report a case of a patient who developed fatal myocardial infarction and acute renal failure secondary to CAPS following an elective splenectomy.

## 2. Case Report

A 79-year-old Indian man was referred to the Division of Hematology, McGill University Health Centre, Montreal, QC, Canada, for isolated thrombocytopenia. His past medical history was limited to mild dyslipidemia, and he was taking no medications. Physical examination was significant for the absence of hemorrhagic manifestations, lymphadenopathy, and splenomegaly. The blood count confirmed mild thrombocytopenia (platelet count of 85 × 10^9^/L) and no evidence of clumping or schistocyte formation. A prolonged prothrombin time (86.5 seconds) with a normal thrombin time led to a complete immune workup, revealing the presence of a circulating lupus anticoagulant antibody detected by a clotting assay (American Diagnostica Inc., Montreal, QC, Canada) in the absence of antiphospholipid antibodies (IgG and IgM). The antinuclear antibody (ANA) test was positive and homogeneous at 1/160, while anti-DNA binding complement was normal. Further laboratory analyses revealed a normal liver and kidney profile. Serology testing for HIV and hepatitis B and C was negative. Bone marrow aspiration and biopsy suggested an elevated number of megakaryocytes compatible with peripheral platelet destruction. Further investigation led to the diagnosis of immune thrombocytopenia (ITP) with an incidental finding of a lupus anticoagulant antibody. In the context of no previous history of a thrombotic event, no anticoagulant treatment was recommended. 

Four years later in February 2009, the patient presented to the Emergency Department with a painful red toe felt to be secondary to arterial thrombosis. This complaint combined with the presence of a persistent positive lupus anticoagulant prompted the diagnosis of antiphospholipid syndrome. The arterial thrombosis improved with low molecular weight heparin (LMWH), and then long-term anticoagulation with warfarin following completed resolution. 

One year later, the patient presented with petechiae on his lower extremities and more severe thrombocytopenia of 10 × 10^9^/L. The lack of response to a combination of prednisone (1 mg/kg/day) and intravenous (IV) immunoglobulin (1 gr/kg/day for 2 consecutive days) after 2 weeks led to the withholding of his anticoagulation, and subsequently, a splenectomy was performed.

His platelet count increased to 50 × 10^9^/L on the first post-operative day, and therapeutic anticoagulation was restarted with LMWH. On the third post-operative day, the patient suffered a myocardial infarct with inferolateral ST depression and an increased serum troponin I to 7.43 *μ*g/L (normal <0.06 *μ*g/L). Due to the persistence of thrombocytopenia (platelet < 50 × 10^9^/L), acetylsalicylic acid and clopidogrel therapy were not initiated. The LMWH was switched to intravenous unfractionated heparin. Regular platelet transfusions were also administered to maintain a platelet count above 50 × 10^9^/L.

 A transthoracic echocardiogram showed a mildly decreased global left ventricular systolic function of 45–50%, moderate mitral regurgitation secondary to restricted systolic leaflet motion, and pulmonary artery systolic pressure of 60 mmHg. 

At 36 hours after the onset of the chest pain, the patient underwent a coronary angiogram, which showed normal coronaries. The serum troponin-I continued to rise up to 52 *μ*g/L, and he developed cardiogenic pulmonary edema requiring mechanical ventilation. In the interim, his condition was complicated by acute renal failure with elevated serum creatinine to 264 *μ*mol/L from a baseline of 127 *μ*mol/L. 

In light of refractory thrombocytopenia with evidence of cardiac ischemia without macrovascular disease and progressive kidney injury, treatment for CAPS was instituted and consisted of daily plasma exchange therapy, IV immunoglobulin (1 g/kg IV on two consecutive days), and pulse steroid treatment (2 mg/kg daily). To minimize the potential risk of heparin-induced thrombocytopenia in the context of preexisting thrombocytopenia in this patient, unfractionated heparin was replaced with argatroban. 

**Figure 1:**
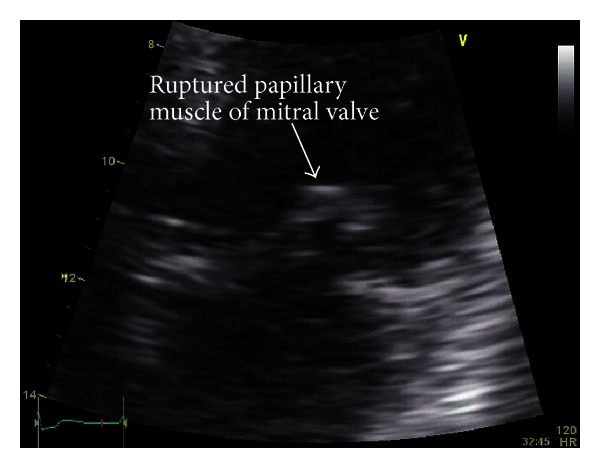
Cardiac echography, apical four chambers view. Echodense prolapsing 10 mm mass attached to the anterior mitral valve leaflet leading to severe mitral regurgitation.

Four days after presentation, the patient developed massive hemoptysis felt to be secondary to the development of sepsis-related disseminated intravascular coagulation (DIC). Argatroban was withheld due to severe bleeding in the context of DIC, and the patient received supportive blood products including cryoprecipitate, fresh frozen plasma, pack red blood cells, and platelets. In the context of refractory thrombocytopenia, rituximab was initiated at the dose of 375 mg/m^2^ IV. Two days later, the patient developed cardiogenic shock, and a repeat transthoracic echocardiogram revealed a new echodense prolapsing mass of 10 mm attached to the anterior mitral valve leaflet, as well as severe mitral regurgitation (Figure 1). His left ventricle was hyperdynamic with an ejection fraction of 65%. Despite intensive care support the patient died from multiorgan failure secondary to systemic hypoperfusion.

**Figure 2:**
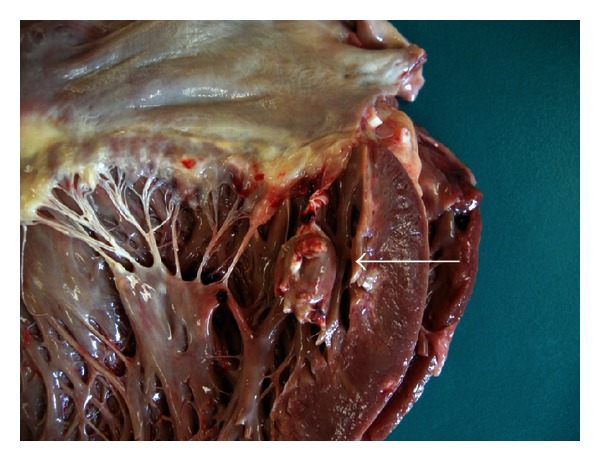
Autopsy image of the mitral valve showing the upper portion of the ruptured papillary muscle (arrow).

 An autopsy showed microvascular thrombi in small myocardial arteries and arterioles consistent with CAPS. Patchy but extensive myocardial necrosis was observed in both ventricles. In the midportion of the anterolateral papillary muscle, this necrosis was most evident and had resulted in rupture (Figure 2). The coronary arteries were normal. Extensive pulmonary hemorrhage and a thromboembolus in the left main pulmonary artery were also observed. There was no evidence of pulmonary microvasculature thrombosis.

## 3. Discussion

This case illustrates dramatic complications from a splenectomy performed in a patient with a recent thrombotic episode on the first toe, laboratory evidence of a lupus anticoagulant, and clinical refractory thrombocytopenia. It reinforces the importance that patients with antiphospholipid antibodies should be closely monitored when they undergo surgical intervention.

Compelling evidence suggests that surgery, trauma, infections, and malignancy can result in a conformational change in the anti-beta2-glycoprotein I antibody complex. This can then interact with endothelial surface receptors such as Annexin A2 and LRP8 and induce proinflammatory and microthrombotic phenomena [1]. Endothelial damage has been reported as the main physiopathological mechanism in CAPS [11–14]. Large venous and arterial vessel occlusions do not dominate the clinical picture in CAPS as opposed to classic APS. Instead, CAPS is characterized by thrombotic occlusion of the microvasculature with liberation of cytokines which lead to further disturbance of vascular endothelial cell (VEC) function and severe multiorgan dysfunction syndrome [11]. VEC injury not only involves regulatory functions within the circulatory, thrombotic, fibrinolytic, neuroendocrine, vasoactive, immune, renal, and respiratory systems but also impacts on the renin-angiotensin system, endothelins (Endothelin-1), natriuretic peptides, prostaglandins, leukotrienes, and dopaminergic and purinergic systems [11]. In this report, CAPS was diagnosed following a coronary angiogram without evidence of macroscopic coronary thrombosis but with clinical evidence of myocardial ischemia and acute renal failure. The rapid initiation of dual therapy with plasmapheresis and argatroban allowed recovery of the patient's platelet count, cardiac markers, and renal function. Due to the paucity of CAPS and its dramatic presentation, no randomized clinical trials have attempted to evaluate the efficacy of therapeutic options and the prognostic factors in the setting of valvular impairment. 

The cessation of anticoagulation after massive hemoptysis in the context of sepsis-related DIC unleashed the prothrombotic cascade despite the addition of rituximab. A repeat cardiac echocardiogram demonstrated the presence of a new prolapsing mass of 10 mm attached to the anterior mitral valve leaflet, and the autopsy report described papillary muscle necrosis at the junction of the mitral valve. 

CAPS, especially in association with refractory immune thrombocytopenia, remains challenging, and all efforts should be made to intervene early with a combination of anticoagulants, immune suppressive therapy, and removal of antibodies by plasma exchange. This case reinforces the role of anticoagulation as one of the main therapeutic agent inhibiting anti-beta-2-glycoprotein I antibody interaction with the endothelium. Other authors have described the use of other pharmacological agents. Defibrotide, an adenosine receptor agonist with affinity to adenosine receptors A1 and A2 and secondary antithrombotic function, has been used as a proposed vascular cell modulator to target VEC injury and to reconstitute multiple VEC functions in a patient with intractable prothrombotic resistance to combined therapy, with effective remission of CAPS [11]. Eculizumab, an anti-C5 monoclonal antibody that blocks activation of terminal complement, was used successfully in other reports [14, 15]. In these studies, terminal complement was shown to be a key innate immune effector engaged by antiphospholipid antibodies to induce thrombophilia, monocyte tissue expression, and leukocyte adhesion to endothelium [14, 15]. For these pharmacological interventions to become accepted as standard therapy, clinical trials will have to be completed.
